# Tumor Suppression and Promotion by Autophagy

**DOI:** 10.1155/2014/603980

**Published:** 2014-09-18

**Authors:** Yenniffer Ávalos, Jimena Canales, Roberto Bravo-Sagua, Alfredo Criollo, Sergio Lavandero, Andrew F. G. Quest

**Affiliations:** ^1^Laboratory of Cellular Communication, Advanced Center for Chronic Diseases (ACCDiS) and Center for Molecular Studies of the Cell, Program in Cell and Molecular Biology, Biomedical Sciences Institute (ICBM), Faculty of Medicine, University of Chile, 8380492 Santiago, Chile; ^2^Laboratory of Molecular Signal Transduction, Advanced Center for Chronic Diseases (ACCDiS) and Center for Molecular Studies of the Cell, Faculty of Chemical and Pharmaceutical Sciences & Faculty of Medicine, University of Chile, 8380492 Santiago, Chile; ^3^Research Institute of Dental Science, Faculty of Dentistry, University of Chile, 8380492 Santiago, Chile; ^4^Department of Internal Medicine, Cardiology Division, University of Texas Southwestern Medical Center, Dallas, TX 75235, USA

## Abstract

Autophagy is a highly regulated catabolic process that involves lysosomal degradation of proteins and organelles, mostly mitochondria, for the maintenance of cellular homeostasis and reduction of metabolic stress. Problems in the execution of this process are linked to different pathological conditions, such as neurodegeneration, aging, and cancer. Many of the proteins that regulate autophagy are either oncogenes or tumor suppressor proteins. Specifically, tumor suppressor genes that negatively regulate mTOR, such as PTEN, AMPK, LKB1, and TSC1/2 stimulate autophagy while, conversely, oncogenes that activate mTOR, such as class I PI3K, Ras, Rheb, and AKT, inhibit autophagy, suggesting that autophagy is a tumor suppressor mechanism. Consistent with this hypothesis, the inhibition of autophagy promotes oxidative stress, genomic instability, and tumorigenesis. Nevertheless, autophagy also functions as a cytoprotective mechanism under stress conditions, including hypoxia and nutrient starvation, that promotes tumor growth and resistance to chemotherapy in established tumors. Here, in this brief review, we will focus the discussion on this ambiguous role of autophagy in the development and progression of cancer.

## 1. Introduction

According to the World Health Organization (WHO), noncommunicable diseases (NCDs) or chronic diseases (CDs), such as cardiovascular diseases, cancer, diabetes, and chronic respiratory diseases, are the leading causes of global mortality. Moreover, because average life-expectation is increasing, their incidence is on the rise and approaching epidemic proportions. The resulting public health burden is spiraling out of control and doing so at an accelerated rate particularly among lower income countries [1]. Despite this rather bleak outlook, the good news is that the impact of these sdiseases could be significantly reduced and considerable suffering avoided by changes in lifestyle to reduce associated risk factors and by the implementation of easy measures for early detection and timely treatment. Specifically, NCDs could be avoided to a considerable extent by reducing four main behavioral risk factors: tobacco use, physical inactivity, harmful use of alcohol, and unhealthy diet. Of interest, particularly in the context of this review series, the latter three risk factors result in a chronic systemic imbalance between caloric intake and consumption, thereby positioning metabolic alterations at the core of chronic disease development. Importantly, although perhaps not as immediately obvious as for diabetes and obesity, cancer is no exception in this respect.

Cancer, a group of diseases generally characterized by abnormal and uncontrolled growth of a population of cells (tumor cells), which eventually invade tissues and form metastases, is one of the leading causes of death worldwide. The latest cancer statistics according to GLOBOCAN 2012 (http://globocan.iarc.fr/Default.aspx) reveal that the global burden of cancer increased in 2012 to 14.1 million new cases and 8.2 million deaths, up from 12.7 million and 7.6 million, respectively, in 2008. Furthermore, these figures are expected to continue increasing to a worrisome 26.4 million new cases and 17 million cancer-related deaths by 2030. In the more developed world (MDW) cancers of the lung, breast, prostate, and colon are the most prevalent types encountered. In contrast, in less developed world (LDW), stomach, liver, oral cavity, and cervical cancers are a more significant concern. These notable differences can be attributed to variations in lifestyles and habits. However, patterns are gradually changing in the LDW and beginning to resemble those of the MDW due to the aging of the population, as well as the acquisition of similar lifestyles and associated risk factors [1, 2].

Thus, despite the many scientific and technological advances that have been developed since the “War on Cancer” was declared by Richard Nixon in 1971, cancer not only remains one of the leading causes of morbidity and mortality worldwide, but it is in fact predicted to become the leading cause of human demise in the coming 20–30 years. In large part, the complexity associated with successful treatment is directly linked to the incredible variety of molecular changes implicated in disease development. The cancer hallmarks defined by Hanahan and Weinberg [3, 4] helped enormously in identifying the general nature of the changes that are required to convert normal cells into tumor cells (transformation). Amongst these, metabolic changes, including the famous Warburg effect, are now recognized as crucial to the development of the transformed phenotype. Bearing this in mind, it should come as no surprise that processes that facilitate cell survival under conditions of metabolic stress are likely to be important in the development of tumors. In this context, we will focus our discussion here on how an evolutionarily ancient response to cellular stress, coined autophagy, may contribute to the pathogenesis of a wide range of cancers. A better understanding of the role of autophagy in tumorigenesis may open up opportunities for more successful treatment of the disease.

## 2. Autophagy: General Aspects and Regulation

Autophagy is a crucial biological process for the survival of unicellular and multicellular eukaryotic organisms under conditions of nutrient deprivation that participates in the maintenance of cellular homeostasis by controlling the quality of proteins and cytoplasmic organelles. The term autophagy (“self-eating”) was introduced by Christian De Duve in the decade of the sixties, based on the observation, by transmission electron microscopy, of double membrane vacuoles containing cytoplasmic material [5]. Nowadays, autophagy is defined as a cellular pathway by which cytoplasmic macromolecules and organelles are delivered to the lysosomes for degradation [6].

At least three different forms of autophagy have been identified to date [6], macroautophagy, microautophagy, and chaperone-mediated autophagy (CMA). These differ with respect to their function and the mode of delivery of the cargo to the lysosomes. In this review, we will focus the discussion on macroautophagy (hereafter referred to as autophagy) and its role in cancer. During macroautophagy, the cargo is sequestered within a* de novo* formed double membrane vesicle, the autophagosome, which fuses with the lysosome to generate autolysosomes, in which lysosomal enzymes degrade the vesicle content. Not surprisingly, autophagy represents an important catabolic mechanism that cancer cells activate in response to cellular stress and/or increased metabolic demands imposed by rapid cell proliferation. In this scenario, autophagy should favor tumor cell survival. Interestingly, however, autophagy also acts as a tumor suppressor mechanism by preventing the accumulation of damaged organelles and proteins. Here, we will discuss our current understanding of the apparently contradictory role that autophagy plays in cancer development and progression.

The autophagosome is the double membrane vesicle that represents the morphological hallmark of autophagy. Autophagosomes originate from the phagophore, an isolation membrane that most likely derives from the endoplasmic reticulum (ER) [7, 8]. However, the source of the membrane still remains a matter of debate and recent findings indicate that both the ER and mitochondria may provide the membranes required [9, 10]. The phagophore then expands and surrounds the material destined for degradation and finally forms the characteristic double membrane vesicle, known as autophagosome. The mature autophagosome then fuses with the lysosome generating the autolysosomes, where the internal membrane and material enclosed in the autolysosome are degraded by the activity of the lysosomal hydrolases and acidification of the luminal microenvironment. The degradation products generated by autophagy are then transferred back to the cytosol by permeases in the autolysosomal membrane and recycled into different metabolic pathways.

The molecular execution of the autophagic pathway—generation, maturation and degradation of the autophagosomes—requires the participation of specific autophagy-related (ATG) proteins [11] that were first described in yeast before orthologs in higher eukaryotes were identified. The ATG proteins organize into multiprotein complexes that function in a nonredundant manner in the different steps of the process. Thus, although many ATGs exist, inhibition of just one ATG suffices to block execution of the autophagic cascade.

In mammalian cells, nucleation of the phagophore is regulated by a protein serine/threonine kinase complex that responds to the mammalian target of rapamycin (mTOR), a key regulator of the autophagic pathway, which shuts down autophagy in the presence of nutrients and growth factors [12]. Phagophore nucleation [7, 13] is regulated by the balance between class I and class III phosphatidylinositol 3-kinase (PI3K) enzymatic activities [14]. The active enzyme VPS34, a class III PI3K, together with the counterparts of yeast Vps15 and Vps30/Atg6, identified in mammals as p150 and Beclin-1, and ATG14 form a PI3K complex that catalyzes the production of phosphatidylinositol-3-phosphate, thereby generating a signal to initiate the recruitment of effectors proteins, such as double FYVE-containing protein 1 (DFCP1) and WD-repeat domain phosphoinositide-interacting (WIPI) family proteins [15–18]. The elongation of the isolation membrane and subsequent closure of the autophagosome require the formation of two ubiquitin-like conjugates. First, ATG12 is conjugated to ATG5 by the sequential activity of ATG7 and ATG10. The resulting ATG5-ATG12 complex interacts with ATG16L, which then oligomerizes to form the ATG16L complex [19]. Second, LC3 (the mammalian homologue of yeast Atg8) is cleaved by the protease ATG4 and then conjugated to the lipid phosphatidylethanolamine via the activity of ATG7 and ATG3 [19, 20]. While the unprocessed form of LC3 (LC3I) is diffusely distributed throughout the cytoplasm, the lipidated form of LC3 (LC3II) specifically accumulates on nascent autophagosomes and thus represents a marker to monitor autophagy [21]. The autophagosome eventually seals off and fuses with lysosomes through mechanisms that remain poorly characterized in mammalian cells [11]. Some regulators of the autophagosome-lysosome fusion process include LC3, the lysosomal proteins LAMP-1 and LAMP-2, the small GTP-binding protein RAB7, and the AAA-type ATPase SKD1 [22–24]. Autophagosome-lysosome fusion then results in the activation of the hydrolases which completely degrade the autophagosomal cargo.

**Figure 1:**
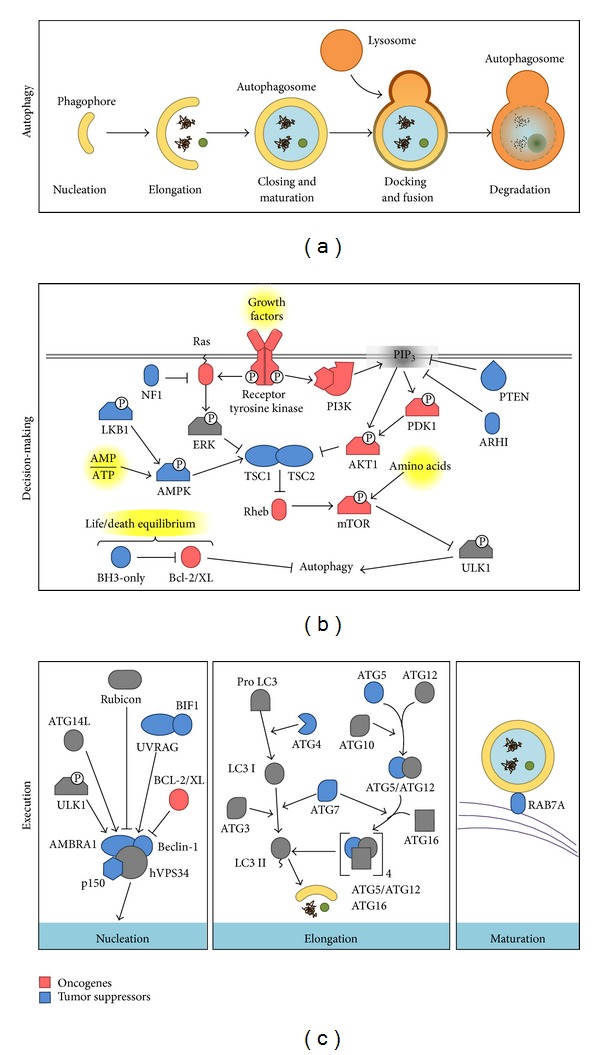
Phases of autophagy and its regulation by oncogenes and tumor suppressors. In (a), the five stages of autophagy are summarized. In (b), inhibition of autophagy by oncogenes (in red) and activation by tumor suppressors (in blue) is shown. Finally, (c) summarizes details of the complex regulation and interplay between different proteins in each stage of autophagy (see text for more details).

Different signaling mechanisms are known to modulate autophagy in mammalian cells [25]. The best characterized pathways are those that modulate autophagy in response to nutritional changes and, as previously mentioned, mTOR is critical for sensing the nutritional status of the cell and regulating the initiation of autophagy [12]. In higher eukaryotes, mTOR can be found in at least two distinct multiprotein complexes, referred to as mTOR complex 1 (mTORC1) and mTOR complex 2 (mTORC2) [26, 27]. The former is considered the principal regulator of autophagy [28]. When nutrients and growth factors are available, mTORC1 inhibits autophagy by phosphorylating and maintaining in an inactive state ULK1, which is required for the formation of the phagophore [29, 30]. As indicated (see Figure 1), mTOR activity is controlled by different signaling pathways triggered via cues from the extracellular and intracellular microenvironment.

AMP-activated protein kinase (AMPK), another sensor of the cellular energy status, responds to decreases in ATP/AMP ratios [31]. In conditions of nutrient deprivation, AMPK directly phosphorylates and inhibits mTOR (not shown in Figure 1). The ensuing reduction in mTOR activity decreases ULK1 phosphorylation and promotes autophagosome formation [31, 32]. Moreover, AMPK also directly activates autophagy by phosphorylation of TSC2 [33, 34]. In summary, autophagy is a highly regulated process that involves a large number of modulators and the complexity of these events will increase as novel components continue to be identified in both mTOR dependent and independent pathways [35].

## 3. Control of Autophagy by Oncogenes and Tumor Suppressors

Most of the proteins that participate in the regulation of autophagy are either tumor suppressor proteins or oncogenes. Perhaps not surprisingly, mechanisms involved in the regulation of autophagy largely overlap with signaling pathways implicated in the control of cancer. Thus, tumor suppressor genes that negatively regulate mTOR, such as PTEN, AMPK, LKB1, and TSC1/2 stimulate autophagy while, conversely, oncogenes that activate mTOR, such as class I PI3K, Ras, RHEB, and AKT, inhibit autophagy [51] (see Table 1). In the following paragraphs, the role of Beclin-1, DAPK, Bcl2/Bcl-XL, and mTOR will be discussed briefly (see Figure 1).

Consistent with this view, Beclin-1, which is part of the class III PI3K complex that promotes autophagy, functions as a tumor suppressor in mammalian cells. Interestingly, monoallelic mutations in the* beclin-1* gene are frequently observed in prostate, ovarian, and breast cancers in humans. In addition, studies in mice have demonstrated that the animals are more sensitive to spontaneous tumor development when* beclin-1* is monoallelically disrupted. These observations provide direct evidence for a role of* beclin-1* as a haploinsufficient tumour suppressor gene implicated in the pathogenesis of several human cancers [41, 52–54]. Additionally, the death-associated protein kinase, DAPK, a protein that phosphorylates Beclin-1 thereby disrupting Beclin-1/BCL-2 complex and favoring autophagy, is another inducer of autophagy that is commonly silenced in different types of human cancers by methylation [55].

BCL-2 and BCL-XL are antiapoptotic members of the BCL-2 family that modulate cell death in an autophagy-independent manner and are overexpressed in several hematological malignancies [56]. There, BCL-2 and BCL-XL suppress cell death and promote survival and growth of cancer cells by suppression of BAK/BAX-dependent pore formation during mitochondrial outer membrane permeabilization (MOMP) [57]. In addition to the role of BCL-2 and BCL-XL in the inhibition of apoptosis, they have also been implicated in oncogenesis as negative regulators of autophagy. Although these proteins do not directly participate in mTOR signaling, they can interact with the Beclin-1 BH3 domain and sequester Beclin-1 into an inactive complex in the ER [58, 59].

The protein kinase mTOR is the major negative regulator of autophagy [60]. This kinase participates in multiple signaling pathways that regulate cell growth, especially downstream of growth factor receptors with tyrosine kinase activity. Interestingly, both the constitutive activation of these receptors, as well as activating mutations of downstream elements in these pathways (Ras, PI3-K, AKT, and PDK-1) or mutations that inactivate negative regulators (TSC1/2, LKB1, and PTEN) are common in the development of cancer [36, 38, 47, 48, 50], suggesting that inhibition of autophagy likely contributes to the onset to tumor development.

## 4. Autophagy as a Tumor Suppressor Mechanism

The first data pointing towards the possible tumor suppressor role of autophagy were obtained in studies of Beclin-1. Monoallelic loss of the* beclin-1* gene on chromosome 17q21 has been reported in 40% to 75% of human breast, ovary, and prostate tumors, suggesting that autophagy represents a tumor suppressor mechanism [41]. Also, a reduction in Beclin-1 protein levels has been observed in various brain cancers [61]. Accordingly, Beclin-1^+/−^ mice have a high incidence of spontaneous tumors, especially lymphoma and hepatocellular carcinoma. Furthermore, the evidence provided suggests that Beclin-1 functions as a haploinsufficient tumor suppressor gene, given that the tumors continued to express Beclin-1 [53, 54]. Moreover, immortalized breast epithelial cells with a monoallelic deletion of Beclin-1 form tumors more rapidly after inoculation into nude mice [62]. More recently, phosphorylation of Beclin-1 on multiple tyrosine residues in an EGFR-dependent manner was found to decrease the activity of the Beclin-1/PI3KC3 complex and therefore decreased autophagy in non-small-cell lung carcinoma cells (NSCLC) and that this effect was reduced in the presence of an inhibitor of EGFR kinase activity. Alternatively, the expression of a tyrosine phosphomimetic mutant of Beclin-1 reduces autophagy and increases tumor growth [63]. Similarly, several proteins that interact with Beclin-1 and positively regulate autophagy, such as AMBRA 1 [64], BIF-1 [45], and UVRAG [65], have been shown to display antiproliferative or tumor suppressor effects. However, a complication here is that all these proteins have other functions that are independent of their role in autophagy, for example in regulating the endocytic pathway [66]. Moreover, the Beclin-1/PI3KC3 complex also controls the ubiquitination and degradation of p53 by regulating the stability and activity of the deubiquitinating enzymes USP13 and USP10 [67]. Given these additional functions, the contribution of such autophagy-independent mechanisms to the observed tumor suppressor phenotype cannot be excluded.

In agreement with the tumor suppressor hypothesis, the generation of knockout mice for specific genes involved in autophagy (ATGs) has shown that defects in specific regulators of this process are associated with the development of a tumorigenic phenotype. Because systemic deletion of* Atg*3,* Atg*5,* Atg*7,* Atg*9, or* Atg*16L1 causes neonatal death [68–72], long-term effects of the inhibition of autophagy could not be assessed until mice with systemic mosaic Atg5 deletion were generated. In this background, systemic mosaic* Atg5* deletion or liver-specific deletion of* Atg7* results in mice that spontaneously develop benign liver adenomas [73]. While these data suggest that defects in autophagy promote the development of benign tumors in this tissue, they also indicate that, in the absence of autophagy, progression to a malignant phenotype is prevented. Similarly, Strohecker et al. showed that the deletion of* Atg7* in mice expressing an activating mutation of B-Raf (*Braf* ^*V600E/+*^) promotes early tumor development in the lung but also inhibits the progression to a more malignant phenotype and increases mouse survival [74]. Additional autophagy-promoting factors that have tumor suppressor functions are Atg4C and RAB7A. For animals deficient in Atg4C, increased susceptibility to the development of fibrosarcomas induced by chemical carcinogens was detected [39]. RAB7A has been shown to prevent growth factor-independent survival by inhibiting cell-autonomous nutrient transporter expression and the* RAB7A* gene is frequently rearranged in different types of leukemia [49, 75].

Despite this evidence that favors a role for autophagy in tumor suppression, some more recent findings concerning Beclin-1 contrast with the previous interpretation of data.* The beclin-1* gene lies close to* BRCA1* on chromosome 17q21 raising the specter that the relevance of the loss of Beclin-1 in ovarian, breast, and prostate cancer may have been overinterpreted. Indeed, deletions encompassing both genes (*BRCA1* and* beclin-1*) and deletions of only* BRCA1* but not* beclin-1* were found in breast and ovarian cancers, which is consistent with* BRCA1* loss representing the primary driver mutation in these cancers. Furthermore, no evidence for* beclin-1* mutations or loss have been detected in any other cancer, which questions whether* beclin-1* is indeed a tumor suppressor in various human cancers [76]. Taken together, the evidence presented supports the hypothesis that autophagy may play an important role in tumor suppression at early stages. However, the findings discussed also reveal the potentially dual nature of this process in tumor development and progression.

### 4.1. Mechanisms Involved in Tumor Suppression by Autophagy

#### 4.1.1. Oxidative Stress and Genomic Instability

One of the most important connections between autophagy and tumor suppression is via the regulation of reactive oxygen species (ROS). Increased ROS production accelerates mutagenesis, increasing the activation of oncogenes, thus stimulating carcinogenesis [77, 78]. Mitochondria are considered the main source of intracellular ROS and their production increases as these organelles age or become damaged [79]. In this context, autophagy helps to avoid damage through selective degradation of defective mitochondria, a process known as mitophagy. Consequently, inhibition of autophagy facilitates genomic instability by promoting the activation of oncogenes [62, 80] and genotoxic effects observed in autophagy-defective cells seem to be dependent on ROS generation [81]. Thus, the selective removal of potentially damaged mitochondria (mitophagy) reduces excessive ROS production and thereby limits tumor-promoting effects dependent on the production of such species [82]. Accordingly, inhibition of autophagy in different models leads to accumulation of defective mitochondria [69, 73, 74, 83–85].

Autophagy also permits the degradation of protein aggregates. Defects in the autophagic process have been associated with the accumulation of protein aggregates and the autophagy substrate p62/SQSTM1. Such events are associated with increased production of ROS, ER stress, and activation of the DNA damage response [81]. The p62 protein is a selective autophagy substrate that accumulates when autophagy is reduced. This scaffolding protein contains a PB1 domain that permits protein oligomerization, an UBA domain required for binding to polyubiquitinated proteins and an LIR domain (LC3-interacting region) necessary for association with LC3. For these reasons, p62 favors selective degradation of both polyubiquitinated proteins and organelles (i.e., mitochondria) [86, 87]. Interestingly, p62 levels are commonly elevated in human tumors. In addition, tumorigenic development observed in autophagy-deficient cells is reversed by genetic inactivation of p62 in various models, suggesting that the accumulation of p62 promotes tumor formation in this context [73, 81, 83, 88]. Moreover, p62 accumulation stabilizes and activates the transcription via NRF-2, by binding to Keap-1, the main negative regulator of NRF-2. In doing so, antioxidant defense is upregulated and may contribute to tumor development [88–90]. Specifically, overexpression of p62 and activation of NRF-2 are critical for anchorage-independent growth observed in hepatocellular carcinoma cells [88].

#### 4.1.2. Inflammation and Necrosis

The tumor microenvironment is defined by complex interactions between various cell types that coexist within tumors (tumor and stromal cells) and crosstalk between these cells regulates both tumor growth and progression. In this context, it is important to note that both inflammatory cells and cytokines are extremely relevant because a proinflammatory environment promotes proliferation and survival of malignant cells, stimulates angiogenesis, metastasis, and modifies the response to drugs [91]. In different models, autophagy inhibition in apoptosis-deficient tumor cells has been shown to promote necrotic cell death, local inflammation, and tumor growth [92]. These results suggest that autophagy may contribute to tumor suppression by restricting tumor necrosis and local inflammation [60]. The anti-inflammatory effect of autophagy has been suggested to be linked to the removal of cell corpses [93] because of findings in* Atg5* ^−/−^ embryonic stem cells, where defects in the clearance of apoptotic bodies during embryonic development are observed [94]. Moreover, a complex connection between autophagy and different aspects of the immune response has been noted, which could contribute to the tumor suppressor role of autophagy, as has been reviewed elsewhere [95].

#### 4.1.3. Autophagic Cell Death and Senescence

Although autophagy is primarily considered a mechanism that permits survival under stress conditions, some reports indicate that, under specific conditions, an increase in autophagic flux may cause cell death due to autophagy and explain in part the tumor suppressor effects [96]. The findings of Pattingre et al. revealed that the expression of a mutant Beclin-1, unable to interact with BCL-2, induced autophagy to a greater extent than wild-type Beclin-1, and unlike the latter, it promoted cell death [58]. More recently, studies in an ovarian cancer cell line showed that ectopic expression of Ras induces autophagic cell death through the upregulation of Beclin-1 and Noxa, a BH3-only protein, which ultimately limits the oncogenic potential of Ras [97]. Similarly, Zhao and colleagues demonstrated that the transcription factor FoxO1 promotes autophagy in a manner independent of its transcriptional activity and induces autophagic cell death in tumor cells, suppressing tumor growth of xenografts in nude mice. These results suggest that autophagy promoted by cytosolic FoxO1 is a tumor suppressor mechanism [98, 99].

Another controversial mechanism that may potentially explain the tumor suppressor activity of autophagy is its role in senescence. Young et al. [100] showed that autophagy is activated during senescence induced by the oncogene Ras in fibroblasts and that inhibition of autophagy in this context delays but does not abrogate the development of the oncogene-mediated senescence phenotype. This finding is important because senescence represents a major intrinsic barrier to malignant transformation [101] although this barrier function may only be transient. In studies of senescence induced by chemotherapy in breast and colon cancer cell lines, autophagy and senescence occur in parallel but not in an interdependent manner. In fact, senescence was only transiently subdued and subsequently recovered despite prolonged inhibition of autophagy [102]. Similar results have been obtained in different experimental settings and are discussed in [103].

## 5. Autophagy as a Promoter of Tumor Growth

### 5.1. Autophagy Promotes Cell Survival under Conditions of Stress

The notion that autophagy represents a mechanism that promotes tumor growth is based on the necessity of tumoral cells to adapt to ischemia in an environment that is hypoxic, as well as growth factor and nutrient deprived. Consistent with this conundrum, autophagy is activated in hypoxic regions of tumors and inhibition of autophagy by monoallelic deletion of* beclin-1* (*Bcn1* ^+/−^) promotes cell death specifically in those regions. These observations suggest a role for autophagy in promoting survival of tumor cells under conditions of metabolic stress [92].

Furthermore, tumor cells generally have high proliferation rates, which translate into higher bioenergetic and biosynthetic needs than is the case for non-transformed cells. These requirements can be satisfied by increasing autophagy as a mechanism that permits obtaining both ATP and metabolic intermediates [66]. Importantly, for tumor cells in which the oncogene Ras is activated, high levels of basal autophagy and dependence on this mechanism for survival are observed [83, 85]. For these reasons, autophagy is thought to promote tumor cell survival by increasing stress tolerance and providing a pathway that permits obtaining the nutrients necessary to meet the enhanced energetic requirements of these cells [66].

### 5.2. Ras-Dependent Tumor Progression and Autophagy Addiction

Small GTPases of the Ras family are involved in signaling pathways important for proliferation, cell survival, and metabolism. RAS-activating mutations are present in 33% of all human cancers, whereby mutations in* KRas* are most prevalent and linked to the development of some of the most lethal cancers, including those of the lung, colon, and pancreas [104, 105]. In human pancreatic cancer, enhanced levels of active autophagy and LC3 correlate with poor patient prognosis [106].* In vitro* studies shown in several cell lines with Ras-activating mutations revealed that these cells exhibit high levels of basal autophagy and marked autophagy-dependent survival under conditions of nutrient deprivation. Moreover, silencing of proteins involved in autophagy promotes the accumulation of dysfunctional mitochondria, low oxygen consumption, and decreased cell growth [83, 85].

*In vivo* studies confirm the aforementioned results. In a KRas-driven pancreatic cancer model, inhibition of autophagy by* Atg5* deletion, decreased the capacity of preneoplastic lesions to progress to invasive cancer, in a manner that was independent of the p53 status [107]. Rosenfeldt et al. reported similar results using a mouse model of humanized pancreatic cancer, but they demonstrated that p53 deletion precludes tumor progression promoted by autophagy. Therefore, the role of autophagy in pancreatic cancer progression may depend on the presence of p53 [108]. In a model of KRas-dependent NSCLC, the inhibition of autophagy through inducible* Atg7* deletion leads to abnormal accumulation of mitochondria and decreases in proliferation and necrotic cell death, which in combination translated into reduced tumor burden. Moreover, in this same study, the absence of Atg7 resulted in progression of Ras-induced adenomas and adenocarcinomas to oncocytomas, benign tumors characterized by accumulation of dysfunctional mitochondria [84]. In studies following up on the role of the Ras pathway in tumor promotion and its dependence on autophagy, Strohecker et al. investigated whether pulmonar carcinogenesis driven by an activating B-Raf mutation was dependent on autophagy.* Atg7* deletion increased oxidative stress and enhanced tumor growth at early stages, but promoted abnormal mitochondria accumulation, proliferation defects, a decrease in tumor burden, and increased survival of animals in more advanced stages of tumorigenesis. Furthermore, when cell lines derived from these tumors were supplemented with glutamine, nutrient deprivation-induced cell death was prevented, suggesting that metabolic stress due to mitochondrial dysfunction may be related to the sensitivity of autophagy-deficient cells to nutrient deprivation [74]. Finally, an* in vitro* study demonstrated that adhesion-independent growth promoted by Ras was dependent on autophagy. Specifically, upon inhibition of autophagy in different cell lines with Ras-activating mutations, the ability of cells to grow in an anchorage-independent manner was lost. These observations underscore the importance of autophagy in maintaining glycolytic activity, which facilitates Ras-mediated anchorage-independent cell growth [109].

### 5.3. Mechanisms Involved in Autophagy Addiction in Ras-Driven Tumors

The dependence on autophagy in Ras-/Raf-driven tumoral cells is explained by the decrease in the acetyl-CoA pool necessary to fuel the tricarboxylic acid (TCA) cycle. Ras activation modulates mitochondrial metabolism by inducing hypoxia-inducible factor (HIF)-1*α*-dependent expression of lactate dehydrogenase (LDH) expression [110], which converts pyruvate to lactate resulting in reduced acetyl-CoA synthesis and, hence, acetyl-CoA depletion. In addition, the Raf/Erk pathway promotes inactivation of LKB1 and subsequently AMPK, thereby preventing *β*-oxidation [111] and decreasing the available mitochondrial acetyl-CoA pool. Due to acetyl-CoA depletion, Ras/Raf-driven tumors require autophagy in order to obtain TCA cycle intermediates. These in turn promote mitochondrial activity, which provides reductive equivalents necessary for oxidative phosphorylation and mitochondrial respiration. Moreover, in Ras-driven tumors, autophagy inhibition promotes the accumulation of dysfunctional mitochondria. This kind of cancer requires autophagy to maintain a pool of functional mitochondria necessary for enhanced energetic requirements of tumoral cells [83, 85].

Beyond the requirement of autophagy for survival of Ras-driven cancer cells, Ras activation also promotes cell signaling events involved in the induction of autophagy by the upregulation of Noxa and Beclin-1 expression [97]. Furthermore, Ras can directly stimulate BNIP3 expression through activation of the Ras/Raf/Erk pathway or indirectly through HIF-1*α* induction [112–114].

### 5.4. Autophagy in Ras-Independent Tumor Progression

The role of autophagy also has been studied in different contexts that are independent of Ras. For instance, in a model of breast cancer driven by the PyMT oncogene, the inhibition of autophagy by FIP200 deletion suppresses mammary tumor initiation and progression. Here, FIP200 ablation increased the number of mitochondria with abnormal morphology in tumor cells and reduced significantly proliferation, but it did not affect apoptosis of mammary tumor cells [115]. Although PyMT requires Ras activation to initiate cell transformation, PI3-kinase and Src activation are also involved [116]. Thus, it would be interesting to determine whether these kinases contribute to dependence on autophagy for cell proliferation. Another study employed a* Palb2* knockout model specific to epithelial breast cells to determine the role of autophagy in breast cancer progression. PALB2 is a protein that cooperates with BRCA1 and BRCA2 in DNA repair via homolog recombination and helps maintain genomic stability.* Palb2* knockout mice develop breast adenocarcinoma when p53 is mutated. Partial inhibition of autophagy by monoallelic loss of Beclin-1 (*Bcn1* ^+/−^) increased apoptosis and delayed tumor growth in a manner dependent on p53. The authors proposed that autophagy promotes tumor growth by p53 suppression when DNA is damaged [117]. These studies indicate that autophagy can promote tumor progression in a manner independent of Ras activation and that autophagy could be a more general mechanism involved in cancer cell survival and tumor progression.

In summary, current evidence points towards autophagy as a mechanism that ensures adequate mitochondrial metabolism in Ras-driven cancers by supplying mitochondrial intermediates via the degradation of macromolecules under basal and starvation conditions [118]. Particularly, Ras-driven tumorigenesis appears to be “addicted to autophagy” for metabolic support and maintenance of rapid tumor growth. All these data explain why autophagy is required in Ras-driven cancers to promote tumor cell survival and tumor progression. Interestingly, however, some more recent evidence indicates that autophagy is also important for tumoral cell survival of other cancers, independent of the Ras activation status.

## 6. Caveolin-1, a Connection to Autophagy?

Caveolin-1 (CAV1) is a scaffolding protein that is essential for caveolae formation, is expressed in a wide variety of tissues, and is involved in many biological processes, including cholesterol homeostasis, vesicular transport, and signal transduction. Moreover, similar to autophagy, CAV1 plays a dual role in cancer, functioning both as a tumor suppressor and promoter of tumor metastasis [119–121]. Although, E-cadherin has been identified as important in determining CAV1 function in this context [122–125], the molecular mechanisms explaining such ambiguous behavior remain largely undefined.

Given the parallels between the roles of CAV1 and autophagy in cancer, it is intriguing to speculate that there might be a connection between the two. Indeed, Martinez-Outschoorn et al. demonstrated, using a coculture system, that CAV1 is degraded via lysosomes in stromal fibroblasts subjected to hypoxia and that this correlated with increased levels of autophagic markers such as LC3, ATG16L, BNIP3, BNIP3L, HIF-1*α*, and NF-kB. Moreover, knockdown of CAV1 in stromal fibroblasts was sufficient to induce the upregulation of lysosomal and autophagic markers, suggesting that the loss of CAV1 in the stromal compartment induces autophagy [126]. Also, loss of CAV1 leads to metabolic reprogramming of stromal cells to support the growth of adjacent tumor cells by delivering energy-rich metabolites and essential building blocks [127]. Consistent with the notion that CAV1 is a negative regulator of autophagy, CAV1 depletion in HCT116 colorectal cancer cells was shown to reduce glucose uptake and ATP production, which then triggered autophagy via activation of AMPK-p53 signaling [128]. Moreover, both* in vitro* cell growth and* in vivo* xenograft tumor growth were attenuated to a greater extent by CAV1 depletion in p53^+/+^ than in p53^−/−^ cells [128].

An inverse relationship between autophagy and CAV1 has also been observed in models of nontransformed cells. For instance, metabolomic profiling of endothelial cell lysates following transfection with si-CAV1 or si-control resulted in marked increases in dipeptide levels for the CAV1 knockdown cells, which was attributed to an increase in autophagy [129]. To corroborate these results, the authors evaluated the processing of LC3 I to LC3 II by western blotting and showed that siRNA-mediated CAV1 knockdown led to an increase in the presence of the autophagy marker LC3-II. Also, treatment with the lysosomal inhibitor bafilomycin A1 markedly increased LC3-II levels, indicating that reduced CAV1 expression leads to an increase in autophagy flux [129]. Recently, CAV1 was also shown to regulate autophagy in cigarette smoking-induced injury of lung epithelium [130]. Specifically, CAV1 depletion increased basal and starvation-induced levels of ATG12-ATG5 and autophagy. Biochemical analysis revealed that CAV1 interacted with ATG5, ATG12, and the active ATG12-ATG5 complex to suppress autophagy in lung epithelial cells, thereby providing new insights as to how CAV1 modulates autophagy in this model [130]. However, details of the molecular mechanisms by which CAV1 regulates autophagy in cancer cells remain to be determined. A rather speculative idea is that the dual role of CAV1 in cancer may be linked to its participation in the control of autophagy. However, further experimentation is required to corroborate this intriguing hypothesis.

In summary, CAV1, a membrane protein typically implicated in the formation of cell surface structures like caveolae and regulation of signalling, also plays a dual role in cancer, functioning as a tumor suppressor at early stages and a tumor promoter later on. The future will reveal how the seemingly opposing roles of autophagy in tumor development and progression are controlled, and to what extent the ambiguous role of CAV1 in cancer may be linked to the control of autophagy.

## 7. Conclusions

**Figure 2:**
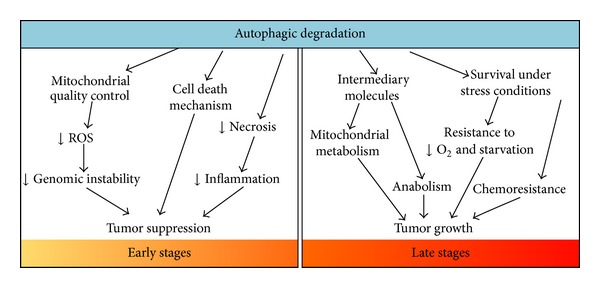
The two facets of autophagy in cancer. At early stages, autophagy acts as a tumor suppressor mechanism by enhancing the degradation of damaged proteins and organelles, mostly mitochondria. In doing so, autophagy acts as a quality control system that decreases ROS production and genomic instability. Moreover, autophagy prevents necrotic cell death in apoptosis-defective cells, thereby reducing local inflammation and tumor growth. Also, autophagy may serve (in some cases) as a mechanism that leads to cell death. On the other hand, at later stages of tumor development, activation of autophagy supplies tumor cells under metabolic stress conditions with nutrients and also maintains mitochondrial metabolism by providing metabolic intermediates, which promote cell survival and tumor growth. Finally, autophagy acts as a mechanism that promotes resistance to cancer therapy.

Autophagy is an evolutionarily conserved mechanism that developed in eukaryotes to ensure protein and organelle homeostasis. A hallmark of cancer cells is their increased proliferation and as a consequence their demand for energy equivalents and specific metabolites, which can be provided by autophagy. In this context, autophagy favors tumor cell development, adaptation, and progression, and particularly some oncogene-driven tumors are “addicted” to autophagy in this respect. However, autophagy also appears to have a tumor suppressor function early in cancer development by eliminating damaged mitochondria and reducing ROS-mediated genotoxic damage (see Figure 2). Accordingly, pharmacological modulation of autophagy in established tumors may represent an important anticancer therapy, as is supported by the use of autophagy inhibitors (chloroquine or hydroxychloroquine) in a large number of clinical trials and currently as a treatment for various kinds of cancers that are generally very aggressive or resistant to therapy (see Table 2).

Alternatively, considering the potential tumor suppressor role of autophagy in early stages of cancer development, one may speculate that stimulation of this process could be useful as a preventive mechanism against the development of cancer. Consistent with this notion, caloric restriction has been shown to prolong life span and reduce cancer incidence in several animal models [131]. Also, treatments with metformin, an activator of the AMPK pathway that stimulates autophagy, are associated with lower risk of different kinds of cancers [132].

Clearly, the role of autophagy in cancer depends on many factors like tissue type, tumor stage, and the type of oncogenic mutation involved. Because of these dramatic differences, more research is required to understand the role of autophagy in cancer biology and how we may harness such knowledge to improve cancer therapies and patient survival.

## Figures and Tables

**Table 1:** Summary of oncogenes and tumor suppressors involved in autophagy regulation.

Oncogenes	Role in autophagy	Evidences of oncogenesis	Reference
AKT1	Upstream inhibitor of autophagy via mTOR activation	Gain-of-function mutations in several cancer types	[36]
BCL-2, BCL-XL	Sequester Beclin-1 into inactive complexes	Overexpressed in several cancer types	[37]
PI3K	Upstream inhibitors of autophagy via AKT1 activation	Gain-of-function mutations in many cancer types	[36, 38]
Ras	Upstream inhibitors of autophagy via mTOR activation	Hyperactivated in several cancer types	[37]

Tumor suppressors	Role in autophagy	Evidences of tumor suppression	

ATG4	Converts LC3 into LC3 I during stress conditions	Mutations in ATG4C increase susceptibility to carcinogens	[39]
ARHI/DIRAS3, PTEN	Relieve autophagy inhibition mediated by PI3K-AKT1	Downregulated in ovarian cancer	[36, 40]
Beclin-1, p150	Required in the nucleation complex for autophagy initiation	Deleted in breast, ovarian, and prostate cancer	[41]
BH3-only proteins	Relieve autophagy inhibition mediated by BCL-2/BCL-XL	Mutated or silenced in many cancer types	[42–44]
UVRAG, BIF1	Positive regulator of the nucleation complex	Deleted or downregulated in colorectal cancer	[45]
DAPK1	Relieve autophagy inhibition mediated by BCL-2/BCL-XL	Silenced in many tumor types	[46]
LKB1/STK11	Promotes autophagy via AMPK activation	Mutated in Peutz-Jeghers syndrome and non-small cell lung carcinomas	[47, 48]
NF1	Relieve autophagy inhibition mediated by Ras	Mutated in neurofibromatosis, juvenile myelomonocytic leukemia	[37]
RAB7A	Modulates endosomal trafficking involved in autophagosome maturation	Rearranged in leukemia, deleted in solid tumors	[49]
TSC1, TSC2	Stimulate Rheb GTPase, thus inhibiting the PI3K-AKT1-mTOR pathway	Mutated in TSC	[50]

**Table 2:** Summary of clinical trials involving autophagy inhibitors (chloroquine or hydroxychloroquine) for cancer treatment (data obtained from http://www.cancer.gov/clinicaltrials).

Cancer type	Therapy	Phase	Status	Protocol ID
Relapsed and refractory multiple myeloma	Cyclophosphamide and pulse dexamethasone with **hydroxychloroquine** or rapamycin	0	Completed	NCT01396200

Glioblastoma multiforme	**Hydroxychloroquine**, radiation, and temozolimide	I, II	Closed	NCT00486603

Pancreas adenocarcinoma	**Hydroxychloroquine**, gemcitabine	I, II	Closed	NCT01128296

Prostate cancer	**Hydroxychloroquine** after prostate cancer treatment	II	Closed	NCT00726596

Non-small cell lung cancer	Erlotinib with or without **hydroxychloroquine**	II	Closed	NCT00977470

Metastatic pancreatic cancer	**Hydroxychloroquine** after prostate cancer treatment	II	Closed	NCT01273805

Relapsed and refractory multiple myeloma	**Chloroquine**, bortezomib, and cyclophosphamide	II	Closed	NCT01438177

Advanced solid tumors irresponsive to chemotherapy	**Hydroxychloroquine**, sunitinib	I	Closed	NCT00813423

B-cell chronic lymphocytic leukemia	**Hydroxychloroquine**	II	Temporarily Closed	NCT00771056

Surgery removable Stage III or Stage IV melanoma	**Hydroxychloroquine**	0	Temporarily Closed	NCT00962845

Relapsed and refractory multiple myeloma	**Hydroxychloroquine**, bortezomib	I, II	Active	NCT00568880

Lung cancer	**Hydroxychloroquine**, gefitinib	I, II	Active	NCT00809237

Ductal carcinoma in situ	**Chloroquine**	I, II	Active	NCT01023477

Colorectal cancer	**Hydroxychloroquine**, folinic acid, 5-fluorouracil, oxaliplatin, and bevacizumab	I, II	Active	NCT01206530

Pancreatic cancer	**Hydroxychloroquine**, protein-bound paclitaxel, and gemcitabine	I, II	Active	NCT01506973

Previously treated renal cell carcinoma	**Hydroxychloroquine**, everolimus	I, II	Active	NCT01510119

Renal cell carcinoma	**Hydroxychloroquine**, aldesleukin	I, II	Active	NCT01550367

Unresectable hepatocellular carcinoma	**Hydroxychloroquine**, transarterial chemoembolization (TACE)	I, II	Active	NCT02013778

Metastatic colorectal cancer	**Hydroxychloroquine**, capecitabine, oxaliplatin, and bevacizumab	II	Active	NCT01006369

Chronic myeloid leukemia	Imatinib mesylate with or without **hydroxychloroquine**	II	Active	NCT01227135

Breast cancer	**Hydroxychloroquine**	II	Active	NCT01292408

Advanced or metastatic breast cancer	**Chloroquine**, taxane	II	Active	NCT01446016

Resectable pancreatic cancer	**Hydroxychloroquine**, capecitabine, and radiation	II	Active	NCT01494155

High grade gliomas	**Hydroxychloroquine**, radiation	II	Active	NCT01602588

Advanced/recurrent non-small cell lung cancer	**Hydroxychloroquine**, paclitaxel, carboplatin, and bevacizumab	II	Active	NCT01649947

Progressive metastatic castrate refractory prostate cancer	Navitoclax, abiraterone acetate with or without **hydroxychloroquine**	II	Active	NCT01828476

Soft tissue sarcoma	**Hydroxychloroquine**, rapamycin	II	Active	NCT01842594

Potentially resectable pancreatic cancer	Protein-bound paclitaxel, gemcitabine with or without **hydroxychloroquine**	II	Active	NCT01978184

Metastatic or unresectable solid tumors	**Hydroxychloroquine**, temozolomide	I	Active	NCT00714181

Irresponsive metastatic solid tumors	**Hydroxychloroquine**, temsirolimus	I	Active	NCT00909831

Stage IV small cell lung cancer	**Chloroquine**	I	Active	NCT00969306

Advanced solid tumors	**Hydroxychloroquine**, vorinostat	I	Active	NCT01023737

Primary renal cell carcinoma	**Hydroxychloroquine** before surgery	I	Active	NCT01144169

Advanced cancer	**Hydroxychloroquine **sirolimus, or vorinostat	I	Active	NCT01266057

Solid tumors	**Hydroxychloroquine**, radiation	I	Active	NCT01417403

Melanoma	**Chloroquine**, radiation, DT01	I	Active	NCT01469455

Advanced solid tumors, melanoma, prostate, or kidney cancer	**Hydroxychloroquine**, Akt inhibitor MK2206	I	Active	NCT01480154

Stages I–III small cell lung cancer	**Chloroquine**, radiation	I	Active	NCT01575782

Refractory or relapsed solid tumors	**Hydroxychloroquine**, sorafenib	I	Active	NCT01634893

Lymphangioleiomyomatosis in women	**Hydroxychloroquine **sirolimus	I	Active	NCT01687179

Relapsed or refractory multiple myeloma	**Hydroxychloroquine**, cyclophosphamide, dexamethasone, and sirolimus	I	Active	NCT01689987

Nonresectable pancreatic adenocarcinoma	**Chloroquine**, gemcitabine	I	Active	NCT01777477

BRAF mutant metastatic melanoma	**Hydroxychloroquine**, vemurafenib	I	Active	NCT01897116

Advanced solid tumors	**Chloroquine**, carboplatin, and gemcitabine	I	Active	NCT02071537

Brain metastasis	**Chloroquine**, radiation	0	Active	NCT01727531

## Abbreviations

AMBRA1: Autophagy/Beclin-1 regulator 1
AMPK: AMP-activated protein kinase
ATG: Autophagy-related
BCL-2: B-cell lymphoma 2
BCL-XL: B-cell lymphoma-extra large
BIF-1: BAX-interacting factor 1
BNIP3: BCL2/adenovirus E1B 19 kDa interacting protein 3
BNIP3L: BCL2/adenovirus E1B 19 kDa interacting protein 3-like
BRCA1: Breast cancer 1, early onset
BRCA2: Breast cancer 2, early onset
ER: Endoplasmic reticulum
FIP200: FAK family-interacting protein of 200 kDa
FoxO: Forkhead box O
LAMP: Lysosomal-associated membrane protein
LC3: Microtubule-associated protein 1 light chain 3 (homolog of yeast Atg8)
LKB1: Liver kinase B1
mTOR: Mammalian target of rapamycin
NF-*κ*B: Nuclear factor kappa-light-chain-enhancer of activated B cells
NRF-2: Nuclear factor erythroid 2-related factor 2
PALB2: Partner and localizer of BRCA2
PI3KC3/VSP34: Phosphatidylinositol 3-kinase, catalytic subunit type 3
PI3K: Phosphatidylinositol 3-kinase
PTEN: Phosphatase and tensin homolog
PyMT: Polyoma middle T-antigen
ULK: Unc-51 like autophagy activating kinase
UVRAG: Ultraviolet radiation resistance-associated gene.
